# Inhibitors of Mitochondrial Dynamics Mediated by Dynamin-Related Protein 1 in Pulmonary Arterial Hypertension

**DOI:** 10.3389/fcell.2022.913904

**Published:** 2022-06-30

**Authors:** Fan Xiao, Rui Zhang, Lan Wang

**Affiliations:** Department of Pulmonary Circulation, Shanghai Pulmonary Hospital, Tongji University School of Medicine, Shanghai, China

**Keywords:** Drp 1, Drp1 inhibitors, mitochondrial dynamics, pulmonary arterial hypertension, mitochondrial fission

## Abstract

Pulmonary arterial hypertension (PAH) is a chronic, lethal pulmonary disease characterized by pulmonary vascular remodeling. It leads to malignant results, such as rupture of pulmonary arterial dissection, dyspnea, right heart failure, and even death. Previous studies have confirmed that one of the main pathological changes of this disease is abnormal mitochondrial dynamics, which include mitochondrial fission, fusion, and autophagy that keep a dynamic balance under certain physiological state. Dynamin-related protein 1 (Drp1), the key molecule in mitochondrial fission, mediates mitochondrial fission while also affecting mitochondrial fusion and autophagy through numerous pathways. There are various abnormalities of Drp1 in PAH pathophysiology, including Drp1 overexpression and activation as well as an upregulation of its outer mitochondrial membrane ligands. These aberrant alterations will eventually induce the development of PAH. With the process of recent studies, the structure and function of Drp1 have been gradually revealed. Meanwhile, inhibitors targeting this pathway have also been discovered. This review aims to shed more light on the mechanism of Drp1 and its inhibitors in the abnormal mitochondrial dynamics of PAH. Furthermore, it seeks to provide more novel insights to clinical therapy.

## Introduction

Pulmonary arterial hypertension (PAH) is a chronic pulmonary disease characterized by abnormal mitochondrial dynamics (Paulin and Michelakis, 2014). According to the 6th World Symposium on PAH, the definition of it is an abnormal elevation of the resting mean pulmonary arterial pressure >20 mmHg with a pulmonary vascular resistance ≥3 Wood Units (WU) (Simonneau et al., 2019). The mitochondria of PAH pulmonary artery smooth muscle cells (PASMCs) suppress glucose oxidation and decrease cytoplasmic glycolysis, inhibiting the Krebs cycle and ETC complexes. Meanwhile, it triggers membrane ion channel inhibition and an increase in intracellular calcium. As a result, the increased calcium as well as the decreased glucose oxidation both directly lead to the PAH PASMCs proliferation (Sutendra and Michelakis, 2014). To validate this process, Jason Boehme’s team created a specific ovine PAH model in which the model PASMCs displayed hyperproliferation after a change in mitochondrial metabolism (Boehme et al., 2016). After that, Paulin and Michelakis (Paulin and Michelakis, 2014) showed that abnormal mitochondrial metabolism is one of the critical mechanisms generating PASMC hyperproliferation in PAH and the central link between mitochondrial metabolism shift and mitochondrial dynamics change. The mitochondrial dynamics include mitochondrial fission, fusion, and autophagy which keep a dynamic balance under a physiological state (Paulin and Michelakis, 2014). In addition, mitochondrial fission accompanies cell division and promotes cell proliferation (Marsboom et al., 2012). As a result, increased mitochondrial fission and decreased mitochondrial fusion and autophagy in PAH promote PASMC mitosis, which leads to PASMC hyperproliferation and apoptosis resistance. Dynamin-related protein 1 (Drp1) is the key molecule in mitochondrial dynamics. It mediates mitochondrial fission while also affecting mitochondrial fusion and autophagy through numerous pathways (Ryan et al., 2015). Drp1 is overexpressed and overactivated in PAH pathophysiology, as well as its outer mitochondrial membrane (OMM) ligands, which bind to and recruit Drp1 to mitochondria. These aberrant alterations will eventually induce the development of PAH (Friedman et al., 2011; Marsboom et al., 2012; Chen et al., 2018; Tian et al., 2018). The structure and function of Drp1 have increasingly been elucidated as research has progressed. At the same time, many inhibitors of pathway targets have been found, like the small molecule GTPase inhibitor, the inhibitor of Drp1 activation, the inhibitor of outer mitochondrial membrane ligand, etc. (Qi et al., 2013; Parra et al., 2017; Joshi et al., 2019; Sunada et al., 2021). Some Drp1 inhibitors have shown significant effects in PAH preclinical and clinical trials, such as restoring mitochondrial function and inhibiting the PAH development (Parra et al., 2017; Tian et al., 2018; Zhuan et al., 2020; Wu et al., 2021). Presently, however, Drp1 inhibitors are mostly discussed in the context of cancer and nervous system disorders, with little clinical research on PAH (Reddy, 2014; Han et al., 2021; Dhapola et al., 2022). This review seeks to shed more light on clinical therapy for PAH and to provide novel points.

## Dynamin-Related Protein 1-Mediated Mitochondrial Homeostasis and Dynamics in Pulmonary Arterial Hypertension

Mitochondria, or highly dynamic organelles, play a key role in cell function. Mitochondria maintain mitochondrial quality control and normal cellular function *via* mitochondrial fission, fusion, and mitophagy. As a result, disordered mitochondrial dynamics lead to numerous diseases (Saito and Sadoshima, 2015). It’s well known that Drp1 plays a major role in mitochondrial fission. Hypoxia or other factors increase Drp1 overexpression in mitochondria, but mitofusin (Mfn) expression, which mediates mitochondrial fusion, decreases. Drp1 promotes mitochondrial fission and mitophagy while inhibiting mitochondrial fusion. PAH will develop as a result of such pathological alterations (Friedman et al., 2011; Parra et al., 2017).

### Mitochondrial Homeostasis and Dynamics

**FIGURE 1:**
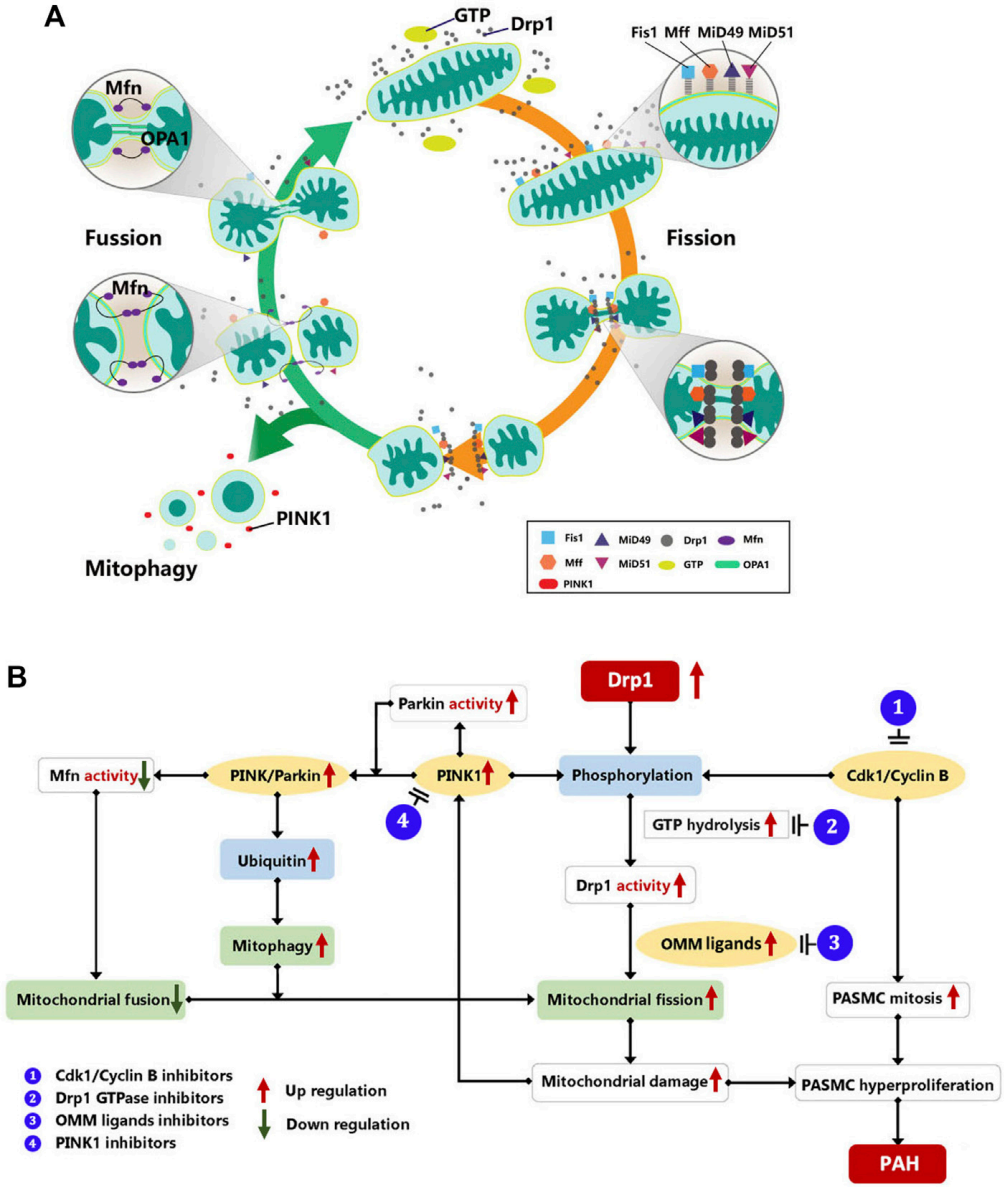
**(A)** Mitochondrial dynamic of mitochondrial homeostasis. Mitochondrial dynamics maintain a balance between mitochondrial fusion and fission in order to ensure that mitochondrial metabolism functions properly. Mitochondrial fusion is regulated by Mfn1 and Mfn2, and OPA1. Mfn primarily mediates OMM fusion, whereas OPA1 requires Mfn1 to mediate mitochondrial inner membrane fusion and control the formation of the mitochondrial crest. On the other hand, mitochondrial fission is mediated by DRP1 and its adapter proteins Fis1, Mff, and MiD49/51. Moreover, faulty mitochondrial fragments are primarily removed by mitophagy mediated by PINK1-Mfn2-Parkin to maintain the quality and function of mitochondria. Drp1, dynamin-related protein 1; FIS1:, mitochondrial fission protein 1; Mff, mitochondrial fission factor; Mfn, mitofusion; MiD49/51, mitochondrial dynamics protein of 49 and 51 kDa; OPA1, optic atrophy protein 1; PINK1, PTEN induced putative kinase 1. **(B)** Drp1 mediate mitochondrial dynamic in PAH. Drp1, dynamin-related protein 1; Mfn, Mitofusin; PINK1, PTEN-induced putative kinase 1; OMM, out mitochondrial membrane; PASMC, pulmonary artery smooth muscle cell; CDK1, cyclin-dependent kinases; PAH, pulmonary artery hypertension.

Mitochondria remove faulty or superfluous components by mitochondrial fission and mitophagy. Meanwhile, mitochondrial biogenesis creates new mitochondria. These procedures help to maintain the mitochondrial quality and function (Saito and Sadoshima, 2015). Initially, mitochondrial fragmentation occurs as a result of mitochondrial damage and a decrease in ATP production. When mitochondrial damage occurs, the AMP-activated protein kinase (AMPK) is immediately activated, causing phosphorylation of the mitochondrial fission factor (Mff), which recruits Drp1 to drive mitochondrial fission (Ingerman et al., 2005; Toyama et al., 2016). Then, faulty mitochondrial fragments are then removed primarily by mitophagy, which is mediated by PTEN induced putative kinase 1 (PINK1)-Parkin-Mfn2. Parkin is recruited to faulty mitochondria and boosts their degradation *via* autophagy (Narendra et al., 2008). Parkin is also controlled by PINK1-dependent phosphorylation, which results in Parkin ubiquitination and activation. At the same time, PINK1 induces Drp1 to be activated. It implies that mitochondrial fission and mitophagy occur simultaneously (Pryde et al., 2016). After the degradation of damaged mitochondria, surplus mitochondrial fragments are fused *via* the regulations of mitofusins (Mfn1 and Mfn2) and optic atrophy protein 1 (OPA1). Mfn primarily mediates OMM fusion, whereas OPA1 requires Mfn1 to mediate mitochondrial inner membrane fusion and control the formation of the mitochondrial crest (Dasgupta et al., 2020). Likewise, PINK/Parkin regulates Mfn post-translational modifications, including phosphorylation and ubiquitination, and suppresses its activation (Gegg et al., 2010; Friedman et al., 2011). The mitochondrial dynamic homeostasis was shown in Figure 1A.

### Dynamin-Related Protein 1-Mediated Mitochondrial Fission in Mitochondrial Dynamics

Mitochondrial fission is vital for maintaining mitochondrial morphology and distribution. In physiological conditions, mitochondrial fission is localized to the mitochondrial midzone (Kleele et al., 2021). This procedure is accompanied by the progress of mitosis. The Drp1-regulated fission burst can evenly distribute organelles into daughter cells (Marsboom et al., 2012). There are two steps involved: mitochondrial contraction and mitochondrial division. Both are considered to be mediated by Drp1 and the classical GTPase dynamic-2 (Dyn2), in which Drp1 plays a major role (Roux et al., 2010; Friedman et al., 2011). On one hand, researchers suggested the endoplasmic reticulum (ER) may be associated with mitochondrial constriction (Friedman et al., 2011). It wraps mitochondria early in the stage of mitochondrial fission. Meanwhile, it determines the fission sites where ER-bound inverted formin 2 (INF2) induces activated Drp1 assembly, resulting in mitochondrial contraction and fission (Friedman et al., 2011; Korobova et al., 2014). Inactive Drp1 is a cytoplasmic monomer, that is, activated by post-translational modification and GTP hydrolysis (Ingerman et al., 2005). Actin filaments would attract activated Drp1 to the fission site *via* a highly dynamic interaction (Hatch et al., 2016). After that, by affecting Drp1/actin interaction characteristics, OMM proteins may access to actin-bound Drp1 and couple with it (Hatch et al., 2016; Kleele et al., 2021). Actin filaments would thus cause early mitochondrial constriction, allowing Drp1-induced subsequent constriction (Korobova et al., 2013). On the other hand, *in vitro* experiments showed that Drp1 just had the function of contracting the membrane (Roux et al., 2010). By comparison with Drp1, early research has demonstrated that Dyn2 promotes membrane division (Lee et al., 2016). However, Fonseca et al. (2019), found that the mitochondrial fission/fusion could proceed normally in mouse fibroblasts with all three mammalian dynamin proteins genes knocked out, as well as cells with knockdown of Dyn2 only (Fonseca et al., 2019). Only Drp1 knockdown inhibitsleads to inhibits mitochondrial fission and increased mitochondrial fusion. It is further proved that Drp1 plays a key role in mitochondrial fission, and Dyn2 protein is not a necessary protein for it.

### Dynamin-Related Protein 1-Mediated Mitochondrial Dynamics in Pulmonary Arterial Hypertension

Under physiological settings, mitochondria not only maintain dynamic homeostasis but also maintain the quality of mitochondria and physiological function *via* mitochondrial fission, fusion, and mitophagy (Sabouny and Shutt, 2020). However, during mitochondrial dysfunction, Drp1 mediates mitochondrial peripheral fission to remove stressed and damaged mitochondria. A recent study (Kleele et al., 2021) demonstrated that the primarily OMM protein that recruited Drp1 became Fis1 in peripheral fission. Additionally, the daughter mitochondria lacked nucleoids and would be degraded by PINK/Parkin-dependent mitophagy (Kleele et al., 2021).

In the PAH models induced by medicines or hypoxia, researchers found an increase in mitochondrial fragments and a decrease in functioning mitochondria stimulated by Drp1 overexpression, which showed as the inhibition of aerobic glucose metabolism and aberrant glycolysis (Boehme et al., 2016; Parra et al., 2017; Tian et al., 2018; Dai et al., 2021). These results could confirm that Drp1 upregulation in PAH enhances mitochondrial fission. Furthermore, in other research, the Drp1 phosphorylation was altered, leading to a rise in Drp1 activity. PAH affected the molecules that regulate Drp1 activity, such as the increase in PINK1 and CDK1/Cyclin B, prompting Drp1 to be more active (Marsboom et al., 2012; Linqing et al., 2021). On one hand, the increased mitosis triggered by CDK1/cyclin B promotes PASMC proliferation and the development of PAH (Pal-Ghosh et al., 2021). On the other hand, upregulated PINK1 promotes PASMC proliferation *via* the PINK1/Parkin-mediated mitotic phagocytosis pathway (Linqing et al., 2021). Simultaneously, increased PINK1 activation can result in Mfn2 phosphorylation and degradation, which inhibits mitochondrial fusion and leads to excessive cell proliferation (Dasgupta et al., 2021). It demonstrates that disrupting mitochondrial homeostasis mediated by increased Drp1 causes the genesis and progression of PAH (Figure 1B).

## Role of Dynamin-Related Protein 1 for Mitochondrial Fission in Pulmonary Arterial Hypertension

As previously stated, Drp1 is the vital factor of mitochondrial fission, which is activated when Drp1 is recruited to the OMM to create a contractile ring (Ingerman et al., 2005). Various factors influence this process, including Drp1 structure, Drp1 activity regulation, and, in particular, OMM proteins that recruit Drp1.

### Structure of Dynamin-Related Protein 1 for Mitochondrial Fission

Drp1, also known as dynamin-like proteins, belongs to the dynamin family (gene Dnm1 in yeast, gene DLP1 in rats, gene Drp1 in nematodes and mammals, etc.) (Praefcke and McMahon, 2004). Dnm1/Drp1 is composed of three components: the polymerization form, the key domain, and its function in mitochondrial division. First, Dnm1/Drp1 occurs in numerous forms in living cells and maintains a dynamic balance between these forms (Strack and Cribbs, 2012). Inactive Dnm1/Drp1 exists in the cytoplasm in a monomer structure (Ingerman et al., 2005). When activated, it self-assembles into dimers, tetramers, and higher-order oligomers. Meanwhile, the Dnm1/Drp1 tetramer is the most common form in the cytoplasm, whereas the oligomer is primarily located in the OMM (Michalska et al., 2018). Second, according to previous studies, Dnm1/Drp1 is like a dynamin in that contains some vital domains: a GTPase domain, a middle assembly domain, a highly variable region (VR), and a GTPase effector domain (GED) (Mears et al., 2011; Strack and Cribbs, 2012) These domains promote Drp1 self-assembly and localization to the OMM (Ingerman et al., 2005; Strack and Cribbs, 2012). Zhu et al. (2004) and his team built up some Drp1 mutants and revealed that the middle assembly domain and GED mainly regulated the intramolecular interaction and promoted the formation of Drp1 oligomers. And they found that the GED is also important in regulating the GTPase domain activity, which could hydrolyze GTP (Zhu et al., 2004). However, the VR acts in a totally different way. It aids Drp1 localization in the OMM by interacting with Drp1’s OMM ligands (Strack and Cribbs, 2012). Finally, as reported in many prior studies, Elena Ingerman proposed that the Dnm1/Drp1 self-assembly works as a switch to trigger mitochondrial constriction and fission (Ingerman et al., 2005). Drp1 oligomers are ring or spiral polymers that bind to OMM ligands and are assembled on the OMM (Ingerman et al., 2005; Strack and Cribbs, 2012). Furthermore, the oligomers’ diameter is suitable for mitochondrial contraction, promoting contraction and fission (Ingerman et al., 2005).

Drp1 level is increased in PAH, enhancing mitochondrial fission and increasing the production of mitochondrial fragments. Parra et al. (2017) built up a chronic-hypoxia-induced cell PAH model (Parra et al., 2017); Tian et al. (2018) built up a monocrotaline (MCT)-induced rat PAH model (Tian et al., 2018). Drp1 overexpression was seen in both models, as was an increase in mitochondrial fragmentation. Meanwhile, the PASMCs in the models displayed hyperproliferation and disordered metabolism. It might be caused by an increase in Drp1 levels, which disrupts the balance of mitochondrial fission/fusion. To put this conclusion to the test, both models were treated with inhibitors or siRNA. The mitochondrial metabolism function was then restored, and PASMC hyperproliferation was inhibited. In addition, the hyperoxia-induced neonatal rat model displayed bronchopulmonary dysplasia, which was followed by PAH (Dai et al., 2021). Meanwhile, they found an increase in Drp1 expression at the beginning of alveolar development in neonatal rats. While the treatment of inhibitors could decrease the level of Drp1 and protect against PAH development. In summary, the Drp1 is a critical factors in the development of PAH. Drp1 inhibition, on the other hand, will result in the reduction of PAH.

### Regulation of Dynamin-Related Protein 1 Activity for Mitochondrial Fission

Drp1 activity is primarily regulated post-translationally by phosphorylation, sumoylation, and ubiquitination (Nakamura et al., 2006; Guo et al., 2017; Han et al., 2020). First of all, phosphorylation is the major regulation of Drp1 activity, which mediates its activation/inactivation. According to previous studies, Drp1’s major phosphorylation sites are Ser585, Ser616, and Ser637, which are primarily regulated by protein kinase A (PKA), PINK1, cyclin dependent kinase 1 (Cdk1)/Cyclin B, Ca^2+^/calmodulin-dependent kinase (CaMKII), Rho-associated coiled-coil-containing protein kinase (ROCK), and other protein kinases (Taguchi et al., 2007; Brand et al., 2018; Han et al., 2020; Ko et al., 2021). Additionally, Cdk1/Cyclin B works as a mitotic initiator, controlling the G2/M phase transition. Therefore, mitochondrial fission accompanies cell-cycle progression from G2 to mitosis. While mitosis is inhibited, the rate of mitochondrial fission would decrease (Marsboom et al., 2012). Secondly, it was demonstrated that Drp1 sumoylation, which is mediated *via* small ubiquitin-like modifier (SUMO) proteins, protects Drp1 from degradation at sites where mitochondrial division occurs. Meanwhile, it may facilitate Drp1 binding with OMM (Wasiak et al., 2007; Guo et al., 2017). For example, it has been found that SUMO-1-ylation of Drp1 increases Drp1’s interaction with the OMM (Wasiak et al., 2007). While Guo et al. (2017) discovered that SUMO-2/3-lyation of Drp1 decreases Drp1’s binding to Mff (Guo et al., 2017). Finally, Drp1 ubiquitination may adversely influence the activity and/or stability of Drp1. Membrane-associated RING Finger Protein 5 (MARCH-V), a new protein in the mitochondria that combines with Mfn2 and Drp1 and promotes Drp1 ubiquitin. Its overexpression leads to a decrease in mitochondrial fission (Nakamura et al., 2006).

In PAH PASMCs, Drp1 activation (phosphorylated on Ser616) is increased. According to Marsboom et al., Drp1 activation in PAH is primarily controlled by Cdk1/Cyclin B, which is increased in PAH (Marsboom et al., 2012). At the same time, since CDK1 is involved in mitosis, the increased mitochondrial fragmentation caused by increased Drp1 activation is accompanied with an increase in PASMC cell proliferation in PAH. As a result, PASMCs make hyperproliferation and becomes resistant to apoptosis (Pal-Ghosh et al., 2021).

### Outer Mitochondrial Membrane Ligand of Dynamin-Related Protein 1 for Mitochondrial Fission

As mentioned above, Drp1 is mainly located on the OMM by binding with OMM ligands. Fis1 was the first OMM ligand found. The appropriate assembly, membrane location, and function of Dnm/Drp1-containing complexes during yeast mitochondrial fission were all dependent on the yeast gene FIS1, which scientists discovered could code the novel OMM protein Fis1 (Mozdy et al., 2000). While Fis1 is less important for mammalian mitochondrial fission, Osellame et al. (2016) confirmed that mitochondrial networks did not change if Fis1 was removed from cells (Osellame et al., 2016). Whereas many studies have found Fis1 is involved in mitophagy and cell apoptosis, a decrease in Fis1 expression would reduce mitophagy and cell death (Lee et al., 2004; Shen et al., 2014). In mammalian cells, Drp1 is recruited to OMM by several ligands, including Fis1, Mff, and MiD49/MiD51. However, any single ligand depletion did not have a significant effect on mitochondrial fission but only on mitochondrial elongation (Loson et al., 2013). On the contrary, overexpression of these ligands would accelerate mitochondrial fission (Loson et al., 2013). When Drp1 recruitment to the mitochondrial surface, Liu and Chan (2015) found Mff is a critical adaptor that selectively associates with higher order oligomers of Drp1 on the OMM. Meanwhile, Mff upregulation causes mitochondrial fission to be elevated (Liu and Chan, 2015). After that, Palmer et al. found that MiD49/MiD51 had more Drp1 recruitment activity than Mff and Fis1, it could recruit both high and low oligomers of Drp1 (Palmer et al., 2011). Moreover, Yu et al. (2021) confirmed that the different assembly state of Drp1 oligomerization in turn affect the aggregation of OMM ligands, and the balance of mitochondrial dynamics (Yu et al., 2021).

In PAH, Drp1 ligand expression is abnormally elevated, promoting mitochondrial fission and drive hyperproliferation and apoptosis resistance (Chen et al., 2018). On the other hand, Joshi et al. (2019) confirmed that mitochondrial fission was primarily mediated by Drp1/Fis1 in a disease condition that favored illness onset. Meanwhile, the inhibitors’ disruption of Drp1 and Fis1 binding may act against the pathogenesis of PAH (Joshi et al., 2019).

## Inhibitors of Dynamin-Related Protein 1 in Pulmonary Arterial Hypertension

According to the different stages of Drp1 in mitochondrial fission, its inhibitors target the appropriate locations, such as the kinase responsible for Drp1 activation; the GTPase domain of Drp1; the OMM ligand combined with Drp1, and so on (Mo et al., 2019; Duan et al., 2020; Sunada et al., 2021). The Drp1 pathway can be inhibited by several inhibitors, which reduce mitochondrial fission and delay or even reverse PAH (Joshi et al., 2019). Inhibitors of Drp1 pathway was listed at Table 1.

**TABLE 1:** Inhibitors of Drp1 pathway.

Pathway		Inhibitor	Experiment objects	Results	References
Drp1 GTPase		Mdivi-1	PAH rats, hypoxia human PASMC	Mitochondrial translocation of Drp1↓, mitochondrial fragmentation↓, ROS↓, mPAP and PVR↓, PASMCs proliferation↓	(Tian et al., 2018; Zhuan et al., 2020; Feng et al., 2021)
		P110	PAH rats, C57BL/6 mice H9C2 cardiomyocytes, neuroblastoma SH 5YSY cells	Drp1 GTPase activity↓, mitochondrial fragmentation↓, ROS↓	(Qi et al., 2013; Luo et al., 2017; Tian et al., 2018; Haileselassie et al., 2019)
		Drpitor1 and Drpitor1a	Human non-small cell lung cancer cell line, xenotransplant mouse model of human lung cancer	Mitochondrial fission↓, cell proliferation↓, cell apoptosis↑, RV diastolic function preserved	Wu et al. (2020)
Drp1 activation	Cdk1/Cyclin B	Ro-3306	Human PAH lungs and PASMC, Human cervical cancer HeLa, human osteosarcoma U2OS and human leukemia HL60 cell lines, rat lung sections and PASMCs	Mitochonrial fission↓, cell proliferation↓, cellular stress ↑, cellular apoptosis↑, RV diastolic function preserved	(Tian et al., 2017; Tian et al., 2018; Sunada et al., 2021)
		Baicalein and baicalein derivatives	MCF-7 tumor cell	CDK1/cyclin B kinase activity↓, cell proliferation↓	Mou et al. (2021)
		Metformin	PAH patients, HeLa cell line, mouse HCC tumor	RV fractional area change↓, pulmonary artery contraction↓, CDK1 expression↓, cell proliferation↓	(Zhou et al., 2018; Liao et al., 2019; Yudhani et al., 2019; Brittain et al., 2020)
	CaMKII	KN-93	Human PAH lungs and PASMC, rat lung sections and PASMCs	Mitochonrial fission↓, Cells proliferation↓, cells migration↓, RV diastolic function preserved	(Tian et al., 2018; Wong et al., 2019; Huetsch et al., 2020; Yang et al., 2021)
		RA-306	Human CaM	CaMKII activity↓, Ser16-PLN phosphorylation↑	Beauverger et al. (2020)
	ROCK	trans-6-((4-aminocyclohexyl)amino)-5-fluoro-2-methoxynicotinamide (Compound 3)	PAH rat	Right ventricle pressure↓, pulmonary vascular remodeling↓	Cavalli et al. (2017)
		Azaindole-1	PAH rat PASMCs, PAH mice	ROCK activity↓, cell proliferation↓, medial wall thickness and muscularisation of peripheral pulmonary arteries↓, right ventricular hypertrophy↓	Dahal et al. (2010)
		H-1337	Human PASMCs, PAH rat	cell proliferation↓, right ventricular, pressure and occlusive vascular lesions↓, RV remodeling↓	Shoji et al. (2021)
		Fasudil	PAH patient	systemic vascular resistance (SVR)↓, pulmonary vascular resistance (PVR)/SVR ratio↓, mean pulmonary arterial pressure (PAP)↓, mean cardiac output↑, mixed venous oxygen saturation↑	(Li et al., 2009; Kojonazarov et al., 2012; Fukumoto et al., 2013; Jiang et al., 2014; Xiao et al., 2015)
		Y-27632	PAH rat	RV hypertrophy↓, pulmonary vascular remodelling↓, hemodynamic parameters promotion	Cantoni et al. (2016)
	PKB	Ganoderic acid A	hypoxia-induced rats’ PASMCs	P-Akt expression↓, PASMCs apoptosis↓, PASMCs proliferation↓	Meng et al. (2022)
		Baicalin	PAH mice	phospho-PKB expression↓, right ventricular systolic pressure↓, hypoxemia improvement	Huang et al. (2017)
Drp1 ligand		SS-31	PAH mice, ICR mice, C57BL/6 mice	Fis1 expression↓, mitochondrial fission↓, LPS-induced cell inflammation↓, cell oxidative stress↓, RV fibrosis↓, pulmonary injury biomarkers↓	(Lu et al., 2016; Zhu et al., 2018; Campbell et al., 2019; Mo et al., 2019)
Other		Trimetazidine (TMZ)	Human hypoxia-induced PASMC	Mitochondrial fission↓, mitochondrial fusion↑, mitochondrial morphology remodeled, PASMC proliferation↓	Parra et al. (2017)

### Inhibitors of Dynamin-Related Protein 1 Activation

As previously stated, Drp1 activation is regulated by serine phosphorylation, which is regulated by Cdk1/Cyclin B, CaMK II, ROCK, and additional kinases. Meanwhile, as the cell cycle progresses from G2 to mitosis, this process is also occurring. According to the findings of Marsboom et al. (2012), it is possible to decrease PASMC hyperproliferation by inhibiting the cell cycle during PAH (Marsboom et al., 2012). As a result, CDK1 inhibitors reduce mitochondrial fission and PAH. RO-3306 is a typical CDK1 inhibitor, and high doses of RO-3306 have been shown to extend and block the G2 phase of mitosis (Sunada et al., 2021). As Paraghamian asserts, RO-3306 inhibits cell proliferation by inducing cell stress and apoptosis, which result in G2 phase arrest (Paraghamian et al., 2020). In summary, by preventing Drp1 activation and inducing cell cycle arrest at G2 phase, RO-3306 could prevent mitochondrial fission and PAH development. On the other hand, KN-93, which is also noteworthy, is the typical inhibitor of CaMKII. By inhibiting CaMK II activation, it may prevent Drp1 phosphorylation and reverse mitochondrial fragmentation (Wong et al., 2019; Yang et al., 2021). *In vitro* studies showed that PASMC growth and migration were inhibited by KN-93 (Huetsch et al., 2020). Marsboom et al. (2012) compared RO-3306 and KN93 (Marsboom et al., 2012). RO-3306 is more effective than KN-93 in lowering intracellular calcium, while KN-93 is more effective at preventing Drp1 phosphorylation.

In addition, ROCK is also the critical molecule in the activation of Drp1. It mediates Drp1 phosphorylation at serine-616 by RhoA, resulting in Drp1 localization at mitochondria (Brand et al., 2018). Moreover, ROCK inhibitors may suppress mitochondrial fission and apoptosis through preventing Drp1 activation (Zhang et al., 2019). Fasudil is one of its typical inhibitors, which has been approved in clinical treatment. In PAH rats, Fasudil and its derivative, Fasudil dichloroacetate, could prevent PAH development by reversing right ventricular systolic pressure (RVSP) and right ventricle hypertrophy index (RVHI), preventing pulmonary vascular remodeling, and also RV hypertrophy and fibrosis, and decreasing inflammatory factor expression (Wenshu et al., 2018; Qi et al., 2019; Liu et al., 2022). In clinical treatment, Fasudil revealed a similar effect in PAH patients that decreased pulmonary vascular resistance PVR and PAP, as well as increased mean cardiac output and mixed venous oxygen saturation (Li et al., 2009; Kojonazarov et al., 2012; Fukumoto et al., 2013; Jiang et al., 2014; Xiao et al., 2015). Furthermore, it could improve the acute hemodynamic changes in CHD-PAH patients (Ruan et al., 2019). The manner of Rho/Rock pathway regulating Drp1 activation showed in Table 1.

### Inhibitors of Dynamin-Related Protein 1 GTPase Activity

Drp1 inhibitors, such as Mdivi-1 and P110 are the Drp1 selective inhibitors which target the GTPase domain (Qi et al., 2013; Duan et al., 2020). Zhuan’s team discovered that Mdivi-1 suppressed mitochondrial fragmentation in hypoxia PASMC and lowered both mPAP and PVR in hypoxic rats (Zhuan et al., 2020). Furthermore, Mdivi-1 treatment of PAH mice reduced right ventricular hypertrophy, the thickness of pulmonary arterioles, muscularized arteries, and PASMCs proliferation in Feng W’s study (Feng et al., 2021). In other researches, both Mdivi-1 and P110 inhibited cell proliferation, promoted mitochondrial fusion, preserved right ventricular (RV) function, and RV reversed ischemia-reperfusion injury (RV-IR) in PAH rats (Tian et al., 2017; Tian et al., 2018). They’re different, though. Mdivi-1’s allosteric and aggregation-blocking effects on Drp1 do not directly influence the Drp1 GTPase domain, but rather DNM2’s (Smith and Gallo, 2017). However, P110 acts directly on the Drp1 GTPase domain. Su et al. showed that it blocked the junction site of Drp1/Fis1, but not on other GTPases. From the part of the function, Mdivi-1 can promote mitochondrial dynamics while also inhibiting the oxygen consumption and ROS production of mitochondrial complex I in a reversible way, a process known as antioxidation (Bordt et al., 2017). This process is independent of Drp1, and does not affect mitochondrial glycolysis and fusion (Dai et al., 2020). P110 inhibits GTPase, preventing Drp1 from moving to the mitochondria, improving mitochondrial structure and function, and decreasing right ventricular diastolic pressure (Tian et al., 2017).

Recently, Wu et al. (2020) discovered the novel GTPase inhibitors, Drbitor1 and Drbitor1 a, which bind to the GTPase domain but had no effect on the Drp1/Fis1 complex. Using RV-IR models, Drpitor1 and Drpitor1a keep the diastolic function of the RV in check. In a recent preclinical study, the researchers (Wu et al., 2021) demonstrated that Drpitor1a inhibits mitochondrial fission and cell proliferation in human PAH PASMC. Meantime, it reduced pulmonary artery medial thickness, prevented RV hypertrophy, and promoted RV function.

### Inhibitors of Dynamin-Related Protein 1 Ligands

Fis1 is the main ligand for Drp1 in pathological conditions. There have been two widely used Fis1 inhibitors. Another inhibitor is the ROS-scavenging mitochondrial antioxidant SS-31, which has been shown in several studies to have an effect on mitochondrial dynamics (Ihenacho et al., 2021). Prior studies have shown that SS-31 may reduce oxidative stress and eliminate ROS (Zhu et al., 2018). Furthermore, it also suppressed apoptosis suppression and remolded mitochondria (Campbell et al., 2019). LPS-stimulated BV-2 cells showed overexpression of Fis1 and more fragmented mitochondrial network. Fis1 levels dropped and mitochondrial fission was avoided after cells were treated with SS-31. In addition, a considerable reduction was also seen in mitochondrial fragmentation (Mo et al., 2019). In a mice model of PAH induced by transverse aortic constriction, SS-31 restored RV fibrosis and pulmonary injury biomarkers. As a result, its treatment efficiently reduces the mice’s PAH progression (Lu et al., 2016).

Drp1 has an essential role in PAH pathogenesis, which is strongly linked to mitochondrial dynamics. It also mediates mitochondrial fission, as well as mitochondrial fusion and mitophagy. The study of Drp1’s various pathways in mitochondrial dynamics (such as Drp1 phosphorylation activation, decomposition of GTP to Drp1 self-assembly, and Drp1 binding ligand localization to mitochondria) can inhibit mitochondrial fission and reverse the process of PAH development by targeting relevant targets and pathways. Inhibitors of Drp1 phosphorylation activation and GTPase are now the focus of a further in-depth investigation. However, other inhibitor targets lack more PAH model experiments. It's an unexplored area in the study of PAH therapy, and additional scientific investigation is needed.

## Abbreviations

AMPK, the activated protein kinase; CaMKII, Ca2+/calmodulin-dependent kinase; Cdk1, cyclin dependent kinase 1; Drp1, dynamin-related protein 1; Dyn2, GTPase dynamic-2; ER, the endoplasmic reticulum; FIS1, mitochondrial fission protein 1; GED, the GTPase effector domain; INF2, inverted formin 2; IR, ischemia-reperfusion injury; Mff, mitochondrial fission factor; Mfn, mitofusin; MiD49/51, mitochondrial dynamics protein of 49 and 51 kDa; OMM, outer mitochondrial membrane; OPA1, optic atrophy protein 1; PAH, pulmonary arterial hypertension; PASMCs, pulmonary artery smooth muscle cells; PINK1, PTEN induced putative kinase 1; PKA, protein kinase A; PKB, protein kinase B; ROCK, Rho-associated coiled-coil-containing protein kinase; RV, right ventricular; RVSP, right ventricular systolic pressure; PVR, pulmonary vascular resistance; SUMO, the small ubiquitin-like modifier; TMZ, trimetazidine; VR, the variable region.

## References

[B1] Beauverger P. Ozoux M.-L. Bégis G. Glénat V. Briand V. Philippo M.-C. (2020). Reversion of Cardiac Dysfunction by a Novel Orally Available Calcium/calmodulin-dependent Protein Kinase II Inhibitor, RA306, in a Genetic Model of Dilated Cardiomyopathy. Cardiovasc. Res. 116 (2), 329–338. 10.1093/cvr/cvz097 31038167

[B2] Boehme J. Sun X. Tormos K. V. Gong W. Kellner M. Datar S. A. (2016). Pulmonary Artery Smooth Muscle Cell Hyperproliferation and Metabolic Shift Triggered by Pulmonary Overcirculation. Am. J. Physiology-Heart Circulatory Physiology 311 (4), H944–H957. 10.1152/ajpheart.00040.2016 PMC5114466 27591215

[B3] Bordt E. A. Clerc P. Roelofs B. A. Saladino A. J. Tretter L. Adam-Vizi V. (2017). The Putative Drp1 Inhibitor Mdivi-1 Is a Reversible Mitochondrial Complex I Inhibitor that Modulates Reactive Oxygen Species. Dev. Cell. 40 (6), 583–594. 10.1016/j.devcel.2017.02.020 28350990 PMC5398851

[B4] Brand C. S. Tan V. P. Brown J. H. Miyamoto S. (2018). RhoA Regulates Drp1 Mediated Mitochondrial Fission through ROCK to Protect Cardiomyocytes. Cell. Signal. 50, 48–57. 10.1016/j.cellsig.2018.06.012 29953931 PMC6361616

[B5] Brittain E. L. Niswender K. Agrawal V. Chen X. Fan R. Pugh M. E. (2020). Mechanistic Phase II Clinical Trial of Metformin in Pulmonary Arterial Hypertension. J. Am. Heart Assoc. 9 (22), e18349. 10.1161/JAHA.120.018349 PMC7763730 33167773

[B6] Campbell M. D. Duan J. Samuelson A. T. Gaffrey M. J. Merrihew G. E. Egertson J. D. (2019). Improving Mitochondrial Function with SS-31 Reverses Age-Related Redox Stress and Improves Exercise Tolerance in Aged Mice. Free Radic. Biol. Med. 134, 268–281. 10.1016/j.freeradbiomed.2018.12.031 30597195 PMC6588449

[B7] Cantoni S. Pastore F. Cavalli S. Marchini G. Villetti G. Nassini R. (2016). Hemodynamic and Anti-remodelling Effect of the Rho Kinase Inhibitor Y-27632 in the Monocrotaline Pulmonary Arterial Hypertension Rat Model. Eur. Respir. J. 48, A5100. 10.1183/13993003.congress-2016.PA5100

[B8] Cavalli S. Cantoni S. Accetta A. Pastore F. Marchini G. Bertolini S. (2017). The Selective Rho Kinase Inhibitor Trans-6-((4-aminocyclohexyl)amino)-5-fluoro-2-methoxynicotinamide Ameliorates Experimental Pulmonary Hypertension. Eur. Respir. J. 50, PA2379. 10.1183/1393003.congress-2017.PA2379

[B9] Chen K.-H. Dasgupta A. Lin J. Potus F. Bonnet S. Iremonger J. (2018). Epigenetic Dysregulation of the Dynamin-Related Protein 1 Binding Partners MiD49 and MiD51 Increases Mitotic Mitochondrial Fission and Promotes Pulmonary Arterial Hypertension. Circulation 138 (3), 287–304. 10.1161/CIRCULATIONAHA.117.031258 29431643 PMC6050130

[B10] Dahal B. K. Kosanovic D. Pamarthi P. K. Sydykov A. Lai Y.-J. Kast R. (2010). Therapeutic Efficacy of Azaindole-1 in Experimental Pulmonary Hypertension. Eur. Respir. J. 36 (4), 808–818. 10.1183/09031936.00140309 20530035

[B11] Dai W. Wang G. Chwa J. Oh M. E. Abeywardana T. Yang Y. (2020). Mitochondrial Division Inhibitor (Mdivi-1) Decreases Oxidative Metabolism in Cancer. Br. J. Cancer. 122 (9), 1288–1297. 10.1038/s41416-020-0778-x 32147668 PMC7188673

[B12] Dai Y. Yu B. Ai D. Yuan L. Wang X. Huo R. (2021). Mitochondrial Fission-Mediated Lung Development in Newborn Rats with Hyperoxia-Induced Bronchopulmonary Dysplasia with Pulmonary Hypertension. Front. Pediatr. 8, 619853. 10.3389/fped.2020.619853 33634054 PMC7902063

[B13] Dasgupta A. Wu D. Tian L. Xiong P. Y. Dunham‐Snary K. J. Chen K. H. (2020). Mitochondria in the Pulmonary Vasculature in Health and Disease: Oxygen‐Sensing, Metabolism, and Dynamics. Compr. Physiol. 10 (2), 713–765. 10.1002/cphy.c190027 32163206

[B14] Dasgupta A. Chen K. H. Lima P. D. A. Mewburn J. Wu D. Al‐Qazazi R. (2021). PINK1‐induced Phosphorylation of Mitofusin 2 at Serine 442 Causes its Proteasomal Degradation and Promotes Cell Proliferation in Lung Cancer and Pulmonary Arterial Hypertension. FASEB J. 35 (8). 10.1096/fj.202100361R PMC8294132 34275172

[B15] Dhapola R. Sarma P. Medhi B. Prakash A. Reddy D. H. (2022). Recent Advances in Molecular Pathways and Therapeutic Implications Targeting Mitochondrial Dysfunction for Alzheimer's Disease. Mol. Neurobiol. 59 (1), 535–555. 10.1007/s12035-021-02612-6 34725778

[B16] Duan C. Wang L. Zhang J. Xiang X. Wu Y. Zhang Z. (2020). Mdivi-1 Attenuates Oxidative Stress and Exerts Vascular Protection in Ischemic/hypoxic Injury by a Mechanism Independent of Drp1 GTPase Activity. Redox Biol. 37, 101706. 10.1016/j.redox.2020.101706 32911435 PMC7490562

[B17] Feng W. Wang J. Yan X. Zhang Q. Chai L. Wang Q. (2021). ERK/Drp1‐dependent Mitochondrial Fission Contributes to HMGB1‐induced Autophagy in Pulmonary Arterial Hypertension. Cell. Prolif. 54 (6), e13048. 10.1111/cpr.13048 33948998 PMC8168414

[B18] Fonseca T. B. Sánchez-Guerrero Á. Milosevic I. Raimundo N. (2019). Mitochondrial Fission Requires DRP1 but Not Dynamins. Nature 570 (7761), E34–E42. 10.1038/s41586-019-1296-y 31217603

[B19] Friedman J. R. Lackner L. L. West M. DiBenedetto J. R. Nunnari J. Voeltz G. K. (2011). ER Tubules Mark Sites of Mitochondrial Division. Science 334 (6054), 358–362. 10.1126/science.1207385 21885730 PMC3366560

[B20] Fukumoto Y. Yamada N. Matsubara H. Mizoguchi M. Uchino K. Yao A. (2013). Double-blind, Placebo-Controlled Clinical Trial with a Rho-Kinase Inhibitor in Pulmonary Arterial Hypertension. Circ. J. 77 (10), 2619–2625. 10.1253/circj.cj-13-0443 23912836

[B21] Gegg M. E. Cooper J. M. Chau K.-Y. Rojo M. Schapira A. H. V. Taanman J.-W. (2010). Mitofusin 1 and Mitofusin 2 Are Ubiquitinated in a PINK1/parkin-dependent Manner upon Induction of Mitophagy. Hum. Mol. Genet. 19 (24), 4861–4870. 10.1093/hmg/ddq419 20871098 PMC3583518

[B22] Guo C. Wilkinson K. A. Evans A. J. Rubin P. P. Henley J. M. (2017). SENP3-mediated deSUMOylation of Drp1 Facilitates Interaction with Mff to Promote Cell Death. Sci. Rep. 7 (1), 43811. 10.1038/srep43811 28262828 PMC5338345

[B23] Haileselassie B. Mukherjee R. Joshi A. U. Napier B. A. Massis L. M. Ostberg N. P. (2019). Drp1/Fis1 Interaction Mediates Mitochondrial Dysfunction in Septic Cardiomyopathy. J. Mol. Cell. Cardiol. 130, 160–169. 10.1016/j.yjmcc.2019.04.006 30981733 PMC6948926

[B24] Han H. Tan J. Wang R. Wan H. He Y. Yan X. (2020). PINK 1 Phosphorylates Drp1 S616 to Regulate Mitophagy‐independent Mitochondrial Dynamics. EMBO Rep. 21 (8), e48686. 10.15252/embr.201948686 32484300 PMC7403662

[B25] Han P. Ren X. Qu X. Meng Y. (2021). The Regulatory Mechanisms of Dynamin-Related Protein 1 in Tumor Development and Therapy. Cancer Biotherapy Radiopharm. 36 (1), 10–17. 10.1089/cbr.2020.3791 32762544

[B26] Hatch A. L. Ji W.-K. Merrill R. A. Strack S. Higgs H. N. (2016). Actin Filaments as Dynamic Reservoirs for Drp1 Recruitment. Mol. Biol. Cell. 27 (20), 3109–3121. 10.1091/mbc.E16-03-0193 27559132 PMC5063618

[B27] Huang X. Wu P. Huang F. Xu M. Chen M. Huang K. (2017). Baicalin Attenuates Chronic Hypoxia-Induced Pulmonary Hypertension *via* Adenosine A2A Receptor-Induced SDF-1/CXCR4/PI3K/AKT Signaling. J. Biomed. Sci. 24 (1), 52. 10.1186/s12929-017-0359-3 28774332 PMC5543745

[B28] Huetsch J. Jiang H. Shimoda L. (2020). CaMKII Is Necessary for Proliferation and Migration of Pulmonary Arterial Smooth Muscle Cells. FASEB J. 34, 1. 10.1096/fasebj.2020.34.s1.03105

[B29] Ihenacho U. K. Meacham K. A. Harwig M. C. Widlansky M. E. Hill R. B. (2021). Mitochondrial Fission Protein 1: Emerging Roles in Organellar Form and Function in Health and Disease. Front. Endocrinol. 12, 660095. 10.3389/fendo.2021.660095 PMC8027123 33841340

[B30] Ingerman E. Perkins E. M. Marino M. Mears J. A. McCaffery J. M. Hinshaw J. E. (2005). Dnm1 Forms Spirals that Are Structurally Tailored to Fit Mitochondria. J. Cell. Biol. 170 (7), 1021–1027. 10.1083/jcb.200506078 16186251 PMC2171542

[B31] Jiang X. Wang Y.-F. Zhao Q.-H. Jiang R. Wu Y. Peng F.-H. (2014). Acute Hemodynamic Response of Infused Fasudil in Patients with Pulmonary Arterial Hypertension: a Randomized, Controlled, Crossover Study. Int. J. Cardiol. 177 (1), 61–65. 10.1016/j.ijcard.2014.09.101 25499341

[B32] Joshi A. U. Ebert A. E. Haileselassie B. Mochly-Rosen D. (2019). Drp1/Fis1-mediated Mitochondrial Fragmentation Leads to Lysosomal Dysfunction in Cardiac Models of Huntington's Disease. J. Mol. Cell. Cardiol. 127, 125–133. 10.1016/j.yjmcc.2018.12.004 30550751 PMC6894172

[B33] Kleele T. Rey T. Winter J. Zaganelli S. Mahecic D. Perreten Lambert H. (2021). Distinct Fission Signatures Predict Mitochondrial Degradation or Biogenesis. Nature 593 (7859), 435–439. 10.1038/s41586-021-03510-6 33953403

[B34] Ko H.-J. Tsai C.-Y. Chiou S.-J. Lai Y.-L. Wang C.-H. Cheng J.-T. (2021). The Phosphorylation Status of Drp1-Ser637 by PKA in Mitochondrial Fission Modulates Mitophagy *via* PINK1/Parkin to Exert Multipolar Spindles Assembly during Mitosis. Biomolecules 11 (3), 424. 10.3390/biom11030424 33805672 PMC7998912

[B35] Kojonazarov B. Myrzaakhmatova A. Sooronbaev T. Ishizaki T. Aldashev A. (2012). Effects of Fasudil in Patients with High-Altitude Pulmonary Hypertension. Eur. Respir. J. 39 (2), 496–498. 10.1183/09031936.00095211 22298615

[B36] Korobova F. Ramabhadran V. Higgs H. N. (2013). An Actin-dependent Step in Mitochondrial Fission Mediated by the ER-Associated Formin INF2. Science 339 (6118), 464–467. 10.1126/science.1228360 23349293 PMC3843506

[B37] Korobova F. Gauvin T. J. Higgs H. N. (2014). A Role for Myosin II in Mammalian Mitochondrial Fission. Curr. Biol. 24 (4), 409–414. 10.1016/j.cub.2013.12.032 24485837 PMC3958938

[B38] Lee Y.-j. Jeong S.-Y. Karbowski M. Smith C. L. Youle R. J. (2004). Roles of the Mammalian Mitochondrial Fission and Fusion Mediators Fis1, Drp1, and Opa1 in Apoptosis. Mol. Biol. Cell. 15 (11), 5001–5011. 10.1091/mbc.e04-04-0294 15356267 PMC524759

[B39] Lee J. E. Westrate L. M. Wu H. Page C. Voeltz G. K. (2016). Multiple Dynamin Family Members Collaborate to Drive Mitochondrial Division. Nature 540 (7631), 139–143. 10.1038/nature20555 27798601 PMC5656044

[B40] Li F. Xia W. Yuan S. Sun R. (2009). Acute Inhibition of Rho-Kinase Attenuates Pulmonary Hypertension in Patients with Congenital Heart Disease. Pediatr. Cardiol. 30 (3), 363–366. 10.1007/s00246-008-9315-z 18953591

[B41] Liao S. Li D. Hui Z. McLachlan C. S. Zhang Y. (2019). Chronic Dosing with Metformin Plus Bosentan Decreases *In Vitro* Pulmonary Artery Contraction from Isolated Arteries in Adults with Pulmonary Hypertension. J. Cardiovasc. Thorac. Res. 11 (3), 189–195. 10.15171/jcvtr.2019.32 31579458 PMC6759611

[B42] Linqing L. Yuhan Q. Erfei L. Yong Q. Dong W. Chengchun T. (2021). Hypoxia-induced PINK1/Parkin-Mediated Mitophagy Promotes Pulmonary Vascular Remodeling. Biochem. Biophysical Res. Commun. 534, 568–575. 10.1016/j.bbrc.2020.11.040 33239167

[B43] Liu H. Pan Z. Ma X. Cui J. Gao J. Miao Q. (2022). ROCK Inhibitor Fasudil Reduces the Expression of Inflammatory Factors in LPS-Induced Rat Pulmonary Microvascular Endothelial Cells *via* ROS/NF-κB Pathway. BMC Pharmacol. Toxicol. 23 (1). 10.1186/s40360-022-00565-7 PMC9013060 35428330

[B44] Liu R. Chan D. C. (2015). The Mitochondrial Fission Receptor Mff Selectively Recruits Oligomerized Drp1. Mol. Biol. Cell. 26 (24), 4466–4477. 10.1091/mbc.E15-08-0591 26446846 PMC4666140

[B45] Losón O. C. Song Z. Chen H. Chan D. C. (2013). Fis1, Mff, MiD49, and MiD51 Mediate Drp1 Recruitment in Mitochondrial Fission. Mol. Biol. Cell. 24 (5), 659–667. 10.1091/mbc.E12-10-0721 23283981 PMC3583668

[B46] Lu H.-i. Huang T.-h. Sung P.-h. Chen Y.-l. Chua S. Chai H.-y. (2016). Administration of Antioxidant Peptide SS-31 Attenuates Transverse Aortic Constriction-Induced Pulmonary Arterial Hypertension in Mice. Acta. Pharmacol. Sin. 37 (5), 589–603. 10.1038/aps.2015.162 27063219 PMC4857546

[B47] Luo F. Herrup K. Qi X. Yang Y. (2017). Inhibition of Drp1 Hyper-Activation Is Protective in Animal Models of Experimental Multiple Sclerosis. Exp. Neurol. 292, 21–34. 10.1016/j.expneurol.2017.02.015 28238799 PMC5484055

[B48] Marsboom G. Toth P. T. Ryan J. J. Hong Z. Wu X. Fang Y.-H. (2012). Dynamin-related Protein 1-mediated Mitochondrial Mitotic Fission Permits Hyperproliferation of Vascular Smooth Muscle Cells and Offers a Novel Therapeutic Target in Pulmonary Hypertension. Circ. Res. 110 (11), 1484–1497. 10.1161/CIRCRESAHA.111.263848 22511751 PMC3539779

[B49] Mears J. A. Lackner L. L. Fang S. Ingerman E. Nunnari J. Hinshaw J. E. (2011). Conformational Changes in Dnm1 Support a Contractile Mechanism for Mitochondrial Fission. Nat. Struct. Mol. Biol. 18 (1), 20–26. 10.1038/nsmb.1949 21170049 PMC3059246

[B50] Meng Y. Ning Q. Liu Y. Pang Y. Ren H. Yang T. (2022). Ganoderic Acid A Suppresses the Phenotypic Modulation of Pulmonary Artery Smooth Muscle Cells through the Inactivation of PI3K/Akt Pathway in Pulmonary Arterial Hypertension. Food Sci. Technol. 42, e83221. 10.1590/fst.83221

[B51] Michalska B. M. Kwapiszewska K. Szczepanowska J. Kalwarczyk T. Patalas-Krawczyk P. Szczepański K. (2018). Insight into the Fission Mechanism by Quantitative Characterization of Drp1 Protein Distribution in the Living Cell. Sci. Rep. 8 (1), 8122. 10.1038/s41598-018-26578-z 29802333 PMC5970238

[B53] Mo Y. Deng S. Zhang L. Huang Y. Li W. Peng Q. (2019). SS-31 Reduces Inflammation and Oxidative Stress through the Inhibition of Fis1 Expression in Lipopolysaccharide-Stimulated Microglia. Biochem. Biophysical Res. Commun. 520 (1), 171–178. 10.1016/j.bbrc.2019.09.077 31582222

[B54] Mou J. Qiu S. Chen D. Deng Y. Tekleab T. (2021). Design, Synthesis, and Primary Activity Assays of Baicalein Derivatives as Cyclin‐dependent Kinase 1 Inhibitors. Chem. Biol. Drug Des. 98 (4), 639–654. 10.1111/cbdd.13917 34233076

[B55] Mozdy A. D. McCaffery J. M. Shaw J. M. (2000). Dnm1p GTPase-Mediated Mitochondrial Fission Is a Multi-step Process Requiring the Novel Integral Membrane Component Fis1p. J. Cell. Biol. 151 (2), 367–380. 10.1083/jcb.151.2.367 11038183 PMC2192649

[B56] Nakamura N. Kimura Y. Tokuda M. Honda S. Hirose S. (2006). MARCH‐V Is a Novel Mitofusin 2‐ and Drp1‐binding Protein Able to Change Mitochondrial Morphology. EMBO Rep. 7 (10), 1019–1022. 10.1038/sj.embor.7400790 16936636 PMC1618377

[B57] Narendra D. Tanaka A. Suen D.-F. Youle R. J. (2008). Parkin Is Recruited Selectively to Impaired Mitochondria and Promotes Their Autophagy. J. Cell. Biol. 183 (5), 795–803. 10.1083/jcb.200809125 19029340 PMC2592826

[B58] Osellame L. D. Singh A. P. Stroud D. A. Palmer C. S. Stojanovski D. Ramachandran R. (2016). Cooperative and Independent Roles of Drp1 Adaptors Mff and MiD49/51 in Mitochondrial Fission. J. Cell. Sci. 129 (11), 2170–2181. 10.1242/jcs.185165 27076521 PMC6919635

[B59] Pal-Ghosh R. Xue D. Warburton R. Hill N. Polgar P. Wilson J. L. (2021). CDC2 Is an Important Driver of Vascular Smooth Muscle Cell Proliferation *via* FOXM1 and PLK1 in Pulmonary Arterial Hypertension. Int. J. Mol. Sci. 22 (13), 6943. 10.3390/ijms22136943 34203295 PMC8268698

[B60] Palmer C. S. Osellame L. D. Laine D. Koutsopoulos O. S. Frazier A. E. Ryan M. T. (2011). MiD49 and MiD51, New Components of the Mitochondrial Fission Machinery. EMBO Rep. 12 (6), 565–573. 10.1038/embor.2011.54 21508961 PMC3128275

[B61] Paraghamian S. E. Huang Y. Hawkins G. M. Fan Y. Yin Y. Zhang X. (2020). The Cdk1 Inhibitor RO3306 Has Anti-tumorigenic Effects in High-Grade Serous Ovarian Cancer. Gynecol. Oncol. 159, 88–89. 10.1016/j.ygyno.2020.05.068 32747013

[B62] Parra V. Bravo-Sagua R. Norambuena-Soto I. Hernández-Fuentes C. P. Gómez-Contreras A. G. Verdejo H. E. (2017). Inhibition of Mitochondrial Fission Prevents Hypoxia-Induced Metabolic Shift and Cellular Proliferation of Pulmonary Arterial Smooth Muscle Cells. Biochimica Biophysica Acta (BBA) - Mol. Basis Dis. 1863 (11), 2891–2903. 10.1016/j.bbadis.2017.07.018 28739174

[B63] Paulin R. Michelakis E. D. (2014). The Metabolic Theory of Pulmonary Arterial Hypertension. Circ. Res. 115 (1), 148–164. 10.1161/CIRCRESAHA.115.301130 24951764

[B64] Praefcke G. J. K. McMahon H. T. (2004). The Dynamin Superfamily: Universal Membrane Tubulation and Fission Molecules? Nat. Rev. Mol. Cell. Biol. 5 (2), 133–147. 10.1038/nrm1313 15040446

[B65] Pryde K. R. Smith H. L. Chau K.-Y. Schapira A. H. V. (2016). PINK1 Disables the Anti-fission Machinery to Segregate Damaged Mitochondria for Mitophagy. J. Cell. Biol. 213 (2), 163–171. 10.1083/jcb.201509003 27091447 PMC5084273

[B66] Qi X. Qvit N. Su Y.-C. Mochly-Rosen D. (2013). Novel Drp1 Inhibitor Diminishes Aberrant Mitochondrial Fission and Neurotoxicity. J. Cell. Sci. 126 (Pt 3), 789–802. 10.1242/jcs.114439 23239023 PMC3619809

[B67] Qi L. Lv T. Cheng Y. Yu M. Han H. Kong H. (2019). Fasudil Dichloroacetate (FDCA), an Orally Available Agent with Potent Therapeutic Efficiency on Monocrotaline-Induced Pulmonary Arterial Hypertension Rats. Bioorg. Med. Chem. Lett. 29 (14), 1812–1818. 10.1016/j.bmcl.2019.05.006 31088713

[B68] Reddy P. H. (2014). Increased Mitochondrial Fission and Neuronal Dysfunction in Huntington's Disease: Implications for Molecular Inhibitors of Excessive Mitochondrial Fission. Drug Discov. Today 19 (7), 951–955. 10.1016/j.drudis.2014.03.020 24681059 PMC4191657

[B69] Roux A. Koster G. Lenz M. Sorre B. Manneville J.-B. Nassoy P. (2010). Membrane Curvature Controls Dynamin Polymerization. Proc. Natl. Acad. Sci. U.S.A. 107 (9), 4141–4146. 10.1073/pnas.0913734107 20160074 PMC2840091

[B70] Ruan H. Zhang Y. Liu R. Yang X. (2019). The Acute Effects of 30 Mg vs. 60 Mg of Intravenous Fasudil on Patients with Congenital Heart Defects and Severe Pulmonary Arterial Hypertension. Congenit. Heart Dis. 14 (4), 645–650. 10.1111/chd.12764 31166081

[B71] Ryan J. Dasgupta A. Huston J. Chen K.-H. Archer S. L. (2015). Mitochondrial Dynamics in Pulmonary Arterial Hypertension. J. Mol. Med. 93 (3), 229–242. 10.1007/s00109-015-1263-5 25672499 PMC4339102

[B72] Sabouny R. Shutt T. E. (2020). Reciprocal Regulation of Mitochondrial Fission and Fusion. Trends Biochem. Sci. 45 (7), 564–577. 10.1016/j.tibs.2020.03.009 32291139

[B73] Saito T. Sadoshima J. (2015). Molecular Mechanisms of Mitochondrial Autophagy/mitophagy in the Heart. Circ. Res. 116 (8), 1477–1490. 10.1161/CIRCRESAHA.116.303790 25858070 PMC4419704

[B74] Shen Q. Yamano K. Head B. P. Kawajiri S. Cheung J. T. M. Wang C. (2014). Mutations in Fis1 Disrupt Orderly Disposal of Defective Mitochondria. Mol. Biol. Cell. 25 (1), 145–159. 10.1091/mbc.E13-09-0525 24196833 PMC3873885

[B75] Shoji H. Yoshida Y. Sanada T. J. Naito A. Maruyama J. Zhang E. (2021). The Isoquinoline-Sulfonamide Compound H-1337 Attenuates SU5416/Hypoxia-Induced Pulmonary Arterial Hypertension in Rats. Cells 11 (1), 66. 10.3390/cells11010066 35011628 PMC8750965

[B76] Simonneau G. Montani D. Celermajer D. S. Denton C. P. Gatzoulis M. A. Krowka M. (2019). Haemodynamic Definitions and Updated Clinical Classification of Pulmonary Hypertension. Eur. Respir. J. 53 (1), 1801913. 10.1183/13993003.01913-2018 30545968 PMC6351336

[B77] Smith G. Gallo G. (2017). To Mdivi-1 or Not to Mdivi-1: Is that the Question? Devel Neurobio 77 (11), 1260–1268. 10.1002/dneu.22519 PMC5654677 28842943

[B78] Strack S. Cribbs J. T. (2012). Allosteric Modulation of Drp1 Mechanoenzyme Assembly and Mitochondrial Fission by the Variable Domain. J. Biol. Chem. 287 (14), 10990–11001. 10.1074/jbc.M112.342105 22334657 PMC3322891

[B79] Sunada S. Saito H. Zhang D. Xu Z. Miki Y. (2021). CDK1 Inhibitor Controls G2/M Phase Transition and Reverses DNA Damage Sensitivity. Biochem. Biophysical Res. Commun. 550, 56–61. 10.1016/j.bbrc.2021.02.117 33684621

[B80] Sutendra G. Michelakis E. D. (2014). The Metabolic Basis of Pulmonary Arterial Hypertension. Cell. Metab. 19 (4), 558–573. 10.1016/j.cmet.2014.01.004 24508506

[B81] Taguchi N. Ishihara N. Jofuku A. Oka T. Mihara K. (2007). Mitotic Phosphorylation of Dynamin-Related GTPase Drp1 Participates in Mitochondrial Fission. J. Biol. Chem. 282 (15), 11521–11529. 10.1074/jbc.M607279200 17301055

[B82] Tian L. Neuber-Hess M. Mewburn J. Dasgupta A. Dunham-Snary K. Wu D. (2017). Ischemia-induced Drp1 and Fis1-Mediated Mitochondrial Fission and Right Ventricular Dysfunction in Pulmonary Hypertension. J. Mol. Med. 95 (4), 381–393. 10.1007/s00109-017-1522-8 28265681 PMC5390778

[B83] Tian L. Potus F. Wu D. Dasgupta A. Chen K.-H. Mewburn J. (2018). Increased Drp1-Mediated Mitochondrial Fission Promotes Proliferation and Collagen Production by Right Ventricular Fibroblasts in Experimental Pulmonary Arterial Hypertension. Front. Physiol. 9, 828. 10.3389/fphys.2018.00828 30042687 PMC6048272

[B84] Toyama E. Q. Herzig S. Courchet J. Lewis T. L. Losón O. C. Hellberg K. (2016). AMP-activated Protein Kinase Mediates Mitochondrial Fission in Response to Energy Stress. Science 351 (6270), 275–281. 10.1126/science.aab4138 26816379 PMC4852862

[B85] Wasiak S. Zunino R. McBride H. M. (2007). Bax/Bak Promote Sumoylation of DRP1 and its Stable Association with Mitochondria during Apoptotic Cell Death. J. Cell. Biol. 177 (3), 439–450. 10.1083/jcb.200610042 17470634 PMC2064824

[B86] Wenshu W. Rui S. Lei Z. (2018). Effect of Fasudil on Pulmonary Vascular Remodeling and Plasma Levels of HIF-1alpha,ET-1 in Rat Model of COPD Complicated with Pulmonary Arterial Hypertension. Chin. Pharmacol. Bull. 34 (11), 1583–1588. 1001-1978(2018)34:11<1583:FSDEDM>2.0.TX;2-H

[B87] Wong M. H. Samal A. B. Lee M. Vlach J. Novikov N. Niedziela-Majka A. (2019). The KN-93 Molecule Inhibits Calcium/Calmodulin-dependent Protein Kinase II (CaMKII) Activity by Binding to Ca2+/CaM. J. Mol. Biol. 431 (7), 1440–1459. 10.1016/j.jmb.2019.02.001 30753871

[B88] Wu D. Dasgupta A. Chen K. H. Neuber‐Hess M. Patel J. Hurst T. E. (2020). Identification of Novel Dynamin‐related Protein 1 (Drp1) GTPase Inhibitors: Therapeutic Potential of Drpitor1 and Drpitor1a in Cancer and Cardiac Ischemia‐reperfusion Injury. FASEB J. 34 (1), 1447–1464. 10.1096/fj.201901467R 31914641

[B89] Wu D. C. Jansen-van Vuuren R. D. Das Gupta A. Chen K. H. Al-Qazazi R. Mewburn J. D. (2021). Pharmacokinetics and Therapeutic Efficacy of Drpitor1a, a Novel Dynamin-Related Protein 1 Inhibitor, in a Preclinical Model of Pulmonary Arterial Hypertension. Circulation 144, A13094. 10.1161/circ.144.suppl1.13094

[B90] Xiao J.-w. Zhu X.-y. Wang Q.-g. Zhang D.-z. Cui C.-S. Zhang P. (2015). Acute Effects of Rho-Kinase Inhibitor Fasudil on Pulmonary Arterial Hypertension in Patients with Congenital Heart Defects. Circ. J. 79 (6), 1342–1348. 10.1253/circj.CJ-14-1015 25797071

[B91] Yang D. Rong R. Yang R. You M. Wang M. Li H. (2021). CaMK II -induced Drp1 Phosphorylation Contributes to Blue Light-Induced AIF-Mediated Necroptosis in Retinal R28 Cells. Biochem. Biophysical Res. Commun. 559, 113–120. 10.1016/j.bbrc.2021.04.082 33940381

[B92] Yu R. Jin S.-B. Ankarcrona M. Lendahl U. Nistér M. Zhao J. (2021). The Molecular Assembly State of Drp1 Controls its Association with the Mitochondrial Recruitment Receptors Mff and MIEF1/2. Front. Cell. Dev. Biol. 9, 706687. 10.3389/fcell.2021.706687 34805137 PMC8602864

[B93] Yudhani R. D. Astuti I. Mustofa M. Indarto D. Muthmainah M. (2019). Metformin Modulates Cyclin D1 and P53 Expression to Inhibit Cell Proliferation and to Induce Apoptosis in Cervical Cancer Cell Lines. Asian Pac J. Cancer Prev. 20 (6), 1667–1673. 10.31557/APJCP.2019.20.6.1667 31244286 PMC7021606

[B94] Zhang Q. Hu C. Huang J. Liu W. Lai W. Leng F. (2019). ROCK1 Induces Dopaminergic Nerve Cell Apoptosis *via* the Activation of Drp1-Mediated Aberrant Mitochondrial Fission in Parkinson's Disease. Exp. Mol. Med. 51 (10), 1–13. 10.1038/s12276-019-0318-z PMC6802738 31578315

[B95] Zhou J. Han S. Qian W. Gu Y. Li X. Yang K. (2018). Metformin Induces miR-378 to Downregulate the CDK1, Leading to Suppression of Cell Proliferation in Hepatocellular Carcinoma. OncoTargets Ther. 11, 4451–4459. 10.2147/OTT.S167614 PMC6074828 30104887

[B96] Zhu P.-P. Patterson A. Stadler J. Seeburg D. P. Sheng M. Blackstone C. (2004). Intra- and Intermolecular Domain Interactions of the C-Terminal GTPase Effector Domain of the Multimeric Dynamin-like GTPase Drp1. J. Biol. Chem. 279 (34), 35967–35974. 10.1074/jbc.M404105200 15208300

[B97] Zhu Y. Wang H. Fang J. Dai W. Zhou J. Wang X. (2018). SS-31 Provides Neuroprotection by Reversing Mitochondrial Dysfunction after Traumatic Brain Injury. Oxidative Med. Cell. Longev. 2018, 1–12. 10.1155/2018/4783602 PMC6129854 30224944

[B98] Zhuan B. Wang X. Wang M.-D. Li Z.-C. Yuan Q. Xie J. (2020). Hypoxia Induces Pulmonary Artery Smooth Muscle Dysfunction through Mitochondrial Fragmentation-Mediated Endoplasmic Reticulum Stress. Aging 12 (23), 23684–23697. 10.18632/aging.103892 33221740 PMC7762493

